# Heart transplantation for adults with congenital heart disease: a single-center experience of 29 years

**DOI:** 10.1186/1749-8090-8-S1-O150

**Published:** 2013-09-11

**Authors:** O Szárszoi, J Pirk, J Maly, M Urban, M Smetana, H Riha, T Kotulak, L Hoskova, I Netuka

**Affiliations:** 1 Department of Cardiac Surgery, Institute for Clinical and Experimental Medicine, Prague, Czech Republic; 2 Department of Cardiac Anaesthesiology, Institute for Clinical and Experimental Medicine, Prague, Czech Republic; 3 Department of Cardiology, Institute for Clinical and Experimental Medicine, Prague, Czech Republic

## Introduction

Advances in palliation of congenital heart disease have resulted in improved survival to adulthood. Many of these patients develop end-stage heart failure requiring heart transplantation. In this study we analyzed the clinical profile and outcome of congenital heart disease patients transplanted at our institution.

## Methods

Of 905 patients who underwent orthotopic heart transplantation at our institute from January 1984 to May 2013, 25 patients ranging in age from 18 to 54 years (18 male), in the end-stage of the heart failure were diagnosed to have congenital heart disease. We retrospectively reviewed the clinical data, operative and postoperative courses of these patients.

## Results

Mean age was 36.2 ± 10.9 years. The main anatomical diagnoses were transposition of the great arteries, single-ventricle defect, tetralogy of Fallot and Ebstein anomaly. Early, 1-, 5 and 10-year survival was 88%, 88%, 73%, 60%, respectively. Operative mortality for the study group was 8 %. There were 2 early deaths, both of which were related to early severe primary graft failure. Four patients from last three years were successfully bridged to heart transplantation with mechanical circulatory support.

## Conclusion

Adult patients with congenital heart disease represent an increasing proportion of heart transplant recipients. Despite high risk surgery because of their complex anatomy, prior surgical palliation, and hemodynamic status, with careful donor and recipient selection, these patients can have excellent early and mid-term survival after heart transplantation.

